# Methyl Jasmonate and Zinc Sulfate Induce Secondary Metabolism and Phenolic Acid Biosynthesis in Barley Seedlings

**DOI:** 10.3390/plants13111512

**Published:** 2024-05-30

**Authors:** Xin Tian, Renjiao Zhang, Zhengfei Yang, Weiming Fang

**Affiliations:** College of Food Science and Engineering, Yangzhou University, Yangzhou 225009, China; dx120230241@stu.yzu.edu.cn (X.T.); mz120201835@stu.yzu.edu.cn (R.Z.); yzf@yzu.edu.cn (Z.Y.)

**Keywords:** phenolic acid, barley seedlings, methyl jasmonate, zinc sulfate

## Abstract

This study aimed to reveal the impact of MeJA and ZnSO_4_ treatments on the physiological metabolism of barley seedlings and the content of phenolic acid. The results showed that MeJA (100 μM) and ZnSO_4_ (4 mM) treatments effectively increased the phenolic acid content by increasing the activities of phenylalanine ammonia-lyase and cinnamate-4-hydroxylase (PAL) and cinnamic acid 4-hydroxylase (C4H) and by up-regulating the expression of genes involved in phenolic acid synthesis. As a result of the MeJA or ZnSO_4_ treatment, the phenolic acid content increased by 35.3% and 30.9% at four days and by 33.8% and 34.5% at six days, respectively, compared to the control. Furthermore, MeJA and ZnSO_4_ treatments significantly increased the malondialdehyde content, causing cell membrane damage and decreasing the fresh weight and seedling length. Barley seedlings responded to MeJA- and ZnSO_4_-induced stress by increasing the activities of antioxidant enzymes and controlling their gene expression levels. Meanwhile, MeJA and ZnSO_4_ treatments significantly upregulated *calcium-adenosine triphosphate*, *calmodulin-dependent protein kinase-related kinase*, and *calmodulin-dependent protein* genes in barley seedlings. This suggested that Ca^2+^ may be the signaling molecule that promotes phenolic acid synthesis under MeJA and ZnSO_4_ treatment. This study deepens the understanding of the phenolic acid enrichment process in barley seedlings under MeJA and ZnSO_4_ treatments.

## 1. Introduction

Phenolic acid is a class of secondary metabolites produced in plants through the phenylpropane pathway and contains a large number of active phenolic hydroxyl groups with strong free radical scavenging ability [1]. Phenolic acid mainly includes ferulic acid, caffeic acid, and coumaric acid. Medical research has found that phenolic acid plays an important role in human health. For example, ferulic acid effectively lowers blood lipid levels [2], caffeic acid lowers blood sugar [3], and coumaric acid alleviates certain cancers [4]. However, the human body cannot synthesize phenolic acid, and it needs to be ingested through external dietary sources [5]. Therefore, the development of foods rich in phenolic acid is becoming increasingly popular worldwide. Barley, as a globally cultivated crop, is rich in proteins, lipids, and various functional components that play an important role in both food and feed production [6]. Germination has been found to stimulate the synthesis of various bioactive substances in barley, including phenolic acid [7,8]. Nowadays, the consumption of sprouted barley products is widespread in many regions, and sprouted barley is used in various foods, such as breakfast products, salads, soups, and baked goods [9,10]. In addition, barley seedlings can be ground into whole-grain flour and added to cereal products, to meet the demands of health-conscious consumers while providing more nutritional benefits [11,12]. Therefore, enriching barley with phenolic acid through sprouting is a promising and cost-effective way to provide consumers with nutritional foods and ingredients.

Studies have shown that abiotic stresses can induce the production of secondary metabolites with defense functions in plants [13]. Therefore, the improvement of secondary metabolite accumulation through abiotic stress has become a major trend in development. In recent years, research has found that zinc salts, especially zinc sulfate (ZnSO_4_), significantly increase phenolic compounds. Tantharapornrerk’s [14] study indicated that exposure to ZnSO_4_ stress inhibited the growth of broccoli sprouts while significantly increasing the total sulfur glucosinolate and total phenolic compound content and their bioactivity. Song et al. [15] found that ZnSO_4_ treatment promoted the production of secondary metabolites in grapes, particularly the synthesis of total phenolics. Furthermore, the combination of zinc oxide and ZnSO_4_ treatment produced peppers with enriched polyphenol content and increased antioxidant activity. However, the impact of ZnSO_4_ treatment on the phenolic acid content in germinating barley has not been documented. Methyl jasmonate (MeJA) is known to be a strong inducer in the signal transduction pathway, promoting the synthesis of secondary metabolites. MeJA has been used to stimulate the production of phenolic in grapes [16]. Wafae Kandoudi et al. [17] found that the phenolic acid content of mint increased significantly as a result of the introduction of MeJA. In addition, exogenous MeJA treatment was found to significantly increase the phenolic acid content of lettuce [18]. These studies indicate that MeJA could effectively induce phenolic acid synthesis. In our previous experimental study, treatment with MeJA or ZnSO_4_ treatments significantly increased the phenolic acid content in barley seedlings, but the mechanism behind phenolic acid enrichment in barley seedlings remains to be explored.

This research examines the impact of MeJA and ZnSO_4_ treatments on phenolic acid synthesis, physiological and biochemical changes, and antioxidant systems in barley seedlings. Furthermore, the study investigated the effects of MeJA or ZnSO_4_ treatments on the expression of crucial genes related to phenolic acid synthesis in barley seedlings, providing insight into the regulatory function of MeJA or ZnSO_4_ in this process. This study serves as a theoretical basis for further investigations into the effects of MeJA or ZnSO_4_ on the growth, development, and synthesis of phenolic acid from barley seedlings.

## 2. Results

### 2.1. Effects of Treatments on Morphology, Seedling Length, Fresh Weight, and Dry Weight

**Figure 1:**
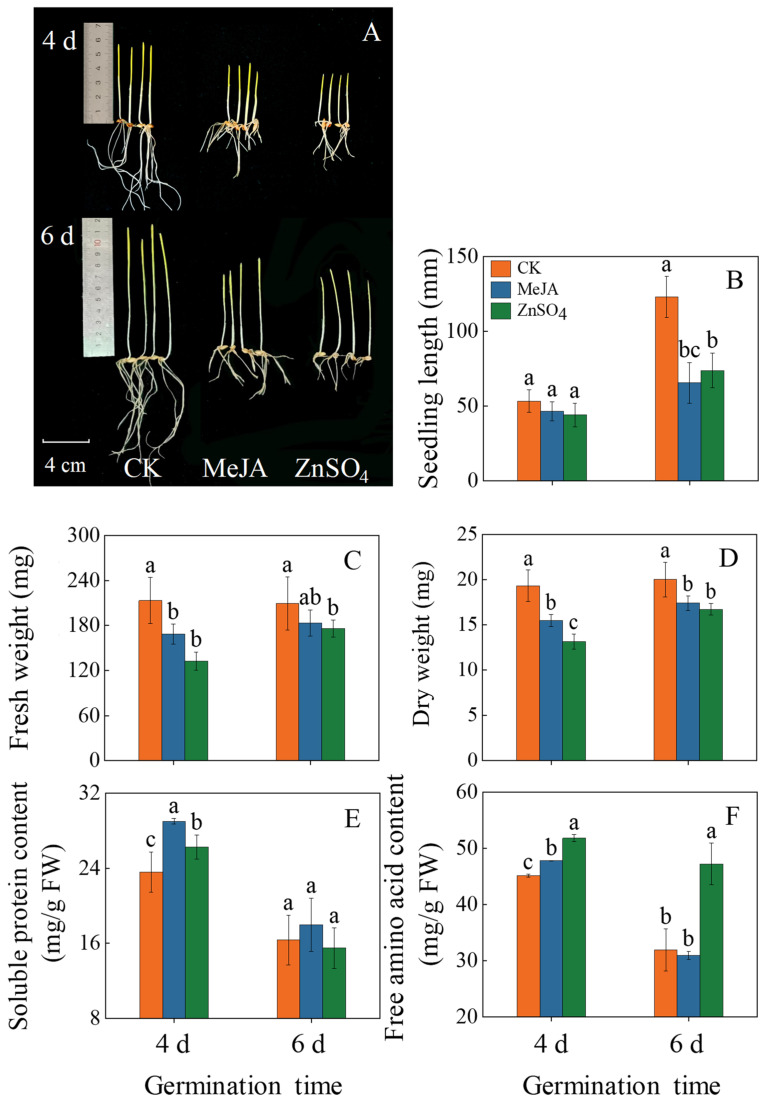
Effects of MeJA/ZnSO_4_ on growth morphology (**A**), seedling length (**B**), fresh weight (**C**), dry weight (**D**), soluble protein content (**E**), and free amino acid content (**F**) of barley seedlings. The error bars indicate the standard deviations of each data point (*n* = 3). Lowercase letters indicate significant differences (*p* < 0.05) between treatments for the same germination time.

Compared to the control, MeJA and ZnSO_4_ treatment inhibited the seedling growth of seedlings on the sixth day (Figure 1A). Moreover, compared to the control, the MeJA and ZnSO_4_ treatments reduced seedlings’ growth by 16.74% and 19.94%, respectively (Figure 1B). On the fourth day of germination, MeJA and ZnSO_4_ treatments markedly decreased the fresh weight and dry weight of seedlings compared to the control (Figure 1C,D, *p* < 0.05). At four days of germination, free amino acid and soluble protein contents in MeJA-treated seedlings were significantly increased by 25.1% and 6.1%, respectively, compared with the control. Meanwhile, ZnSO_4_ treatment significantly increased the soluble protein content in six-day seedlings (Figure 1E, *p* < 0.05).

### 2.2. Effects of Treatments on Total Phenolic and Phenolic Acid Content

**Figure 2:**
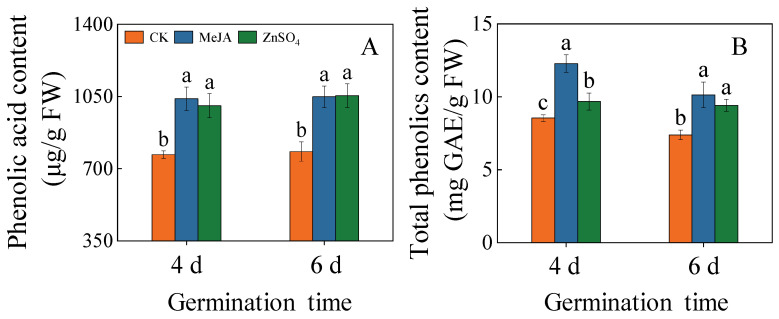
Effects of MeJA/ZnSO_4_ on the phenolic acid content (**A**) and the total phenolics content (**B**) of barley seedlings. Lowercase letters indicate significant differences (*p* < 0.05) between treatments for the same germination time.

The total phenolic acid content of MeJA or ZnSO_4_ treatment was significantly increased (Figure 2A, *p* < 0.05). Compared to the control, the phenolic acid content of the 4th-day barley seedlings treated with MeJA and ZnSO_4_ was 1.35 and 1.31 times higher, respectively. In six days, barley seedlings treated with MeJA and ZnSO_4_ exhibited phenolic acid contents that were 1.28 and 1.30 times higher than those of the control, respectively (Figure 2A).

The total phenolic content of the barley seedlings increased dramatically using MeJA and ZnSO_4_ treatments (Figure 2B, *p* < 0.05). Under MeJA treatment, it reached the highest value of 1.21 times the control on germination day four. The above findings showed that MeJA or ZnSO_4_ treatments promoted the accumulation of total phenolics and phenolic acid.

### 2.3. Effects of Treatments on Malondialdehyde (MDA) Content

**Figure 3:**
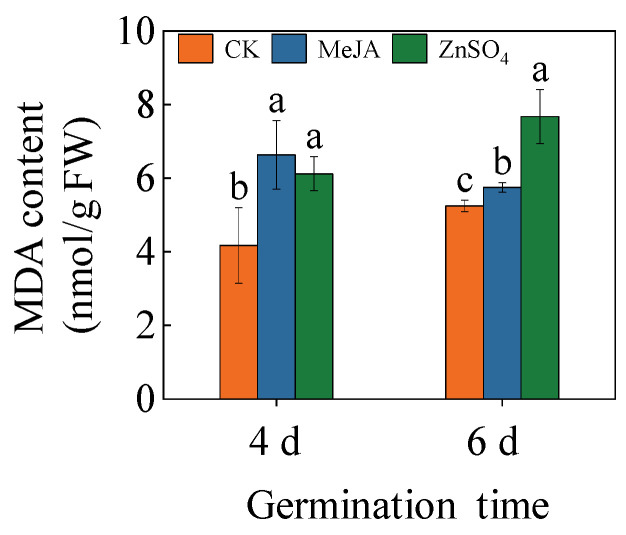
Effects of MeJA/ZnSO_4_ on the MDA content of barley seedlings. Lowercase letters indicate significant differences (*p* < 0.05) between treatments for the same germination time.

The MeJA and ZnSO_4_ treatments significantly increased the MDA content in the sprouts during germination compared to the control (*p* < 0.05). At 4 days of germination, the MDA content was 1.53 and 1.47 times higher than that of the control with the MeJA and ZnSO_4_ treatments, respectively (Figure 3). At 6 days of germination, the MDA content of the MeJA and ZnSO_4_ treatments were 1.10 and 1.46 times higher than the control, respectively. The results reflected that MeJA and ZnSO_4_ treatments disrupted the integrity of the cell membrane.

### 2.4. Effects of Treatments on Key Phenolic Acid Metabolizing Enzymes and Gene Expression

**Figure 4:**
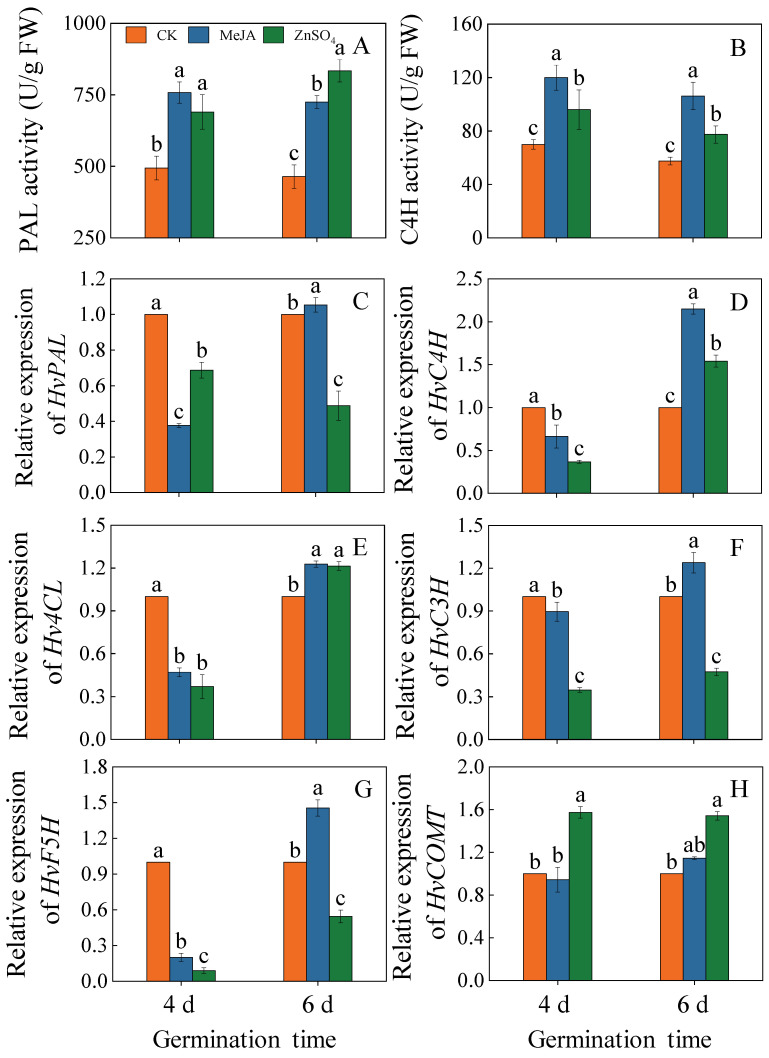
Effects of MeJA/ZnSO_4_ on PAL activity (**A**), C4H activity (**B**), and the gene expression levels of *HvPAL* (**C**), *HvC4H* (**D**), *Hv4CL* (**E**), *HvC3H* (**F**), *HvF5H* (**G**), and *Hv COMT* (**H**) of barley seedlings. Lowercase letters indicate significant differences (*p* < 0.05) between treatments for the same germination time.

PAL and C4H activities increased significantly in barley seedlings treated with MeJA and ZnSO_4_ (Figure 4A,B, *p* < 0.05) compared to the control. Four days after germination, PAL and C4H activities in MeJA-treated barley seedlings reached maximum values of 758.0 U/g and 120.0 U/g, which were 1.53 and 1.71 times the control, respectively. Six days after germination, PAL and C4H activities reached maximum values of 834.0 U/g and 134.3 U/g under ZnSO_4_ treatment, which were 1.80 and 1.85 times the control, respectively.

Compared to the control, the gene expression levels of *HvPAL*, *HvC4H*, *Hv4CL*, *HvC3H*, and *HvF5H* in six-day-old seedlings were significantly up-regulated under MeJA treatment (Figure 4C–H, *p* < 0.05) and were 1.05-, 2.15-, 1.23-, 1.24-, and 1.46-fold higher than the control values, respectively. In six-day-old barley seedlings treated with ZnSO_4_, the gene expression levels of *HvC4H*, *Hv4CL*, and *HvCOMT* were significantly up-regulated (*p* < 0.05) compared to the control, and the expression levels were 1.54, 1.21, and 1.54 times higher than those of the control, respectively.

### 2.5. Effects of Treatments on the Antioxidant Enzyme System and Relative Gene Expression Levels

**Figure 5:**
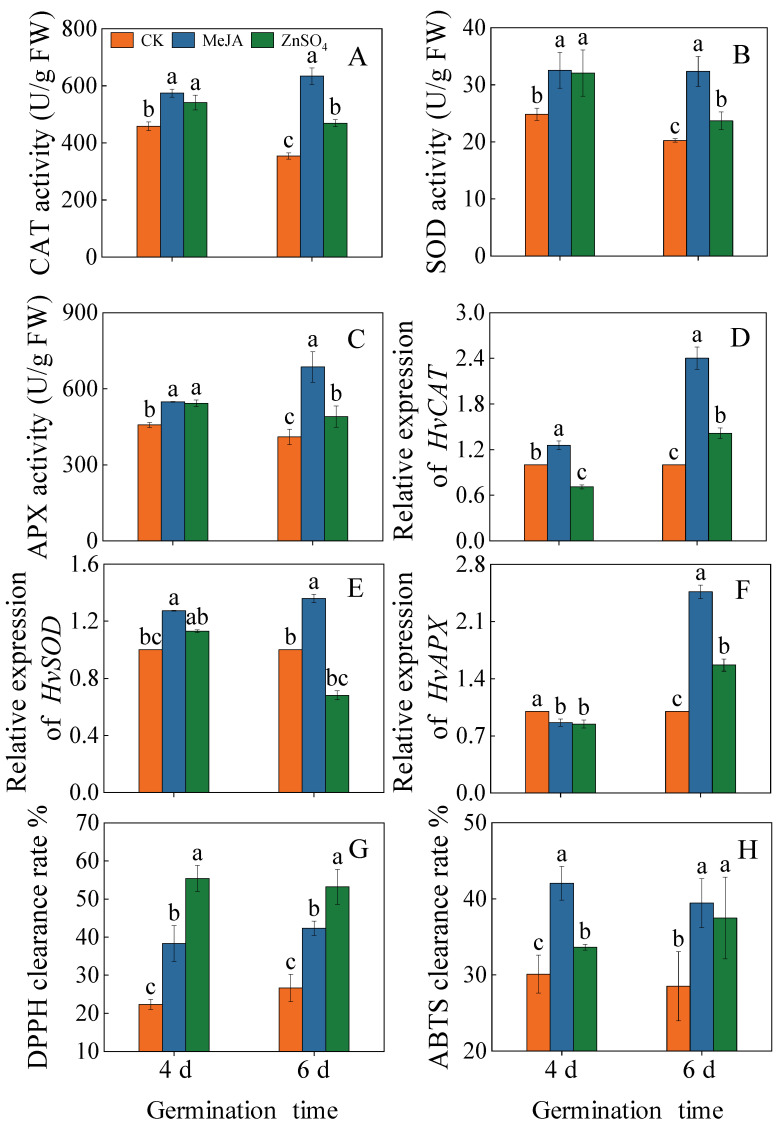
Effects of MeJA/ZnSO_4_ on the enzyme activity of CAT (**A**), SOD (**B**), and APX (**C**) and the gene expression levels of *HvCAT* (**D**), *HvSOD* (**E**), and *HvAPX* (**F**) of barley seedlings. The clearance rates of DPPH (**G**) and ABTS (**H**) of barley seedlings were measured. Lowercase letters indicate significant differences (*p* < 0.05) between treatments for the same germination time.

As shown in Figure 5A–C, the catalase (CAT), superoxide dismutase (SOD), and ascorbic acid peroxidase (APX) activities under the MeJA and ZnSO_4_ treatments were significantly increased (*p* < 0.05). Among them, the highest activities of CAT, SOD, and APX were 634.02 U/g, 32.35 U/g, and 685.83 U/g in six-day-old barley seedlings treated with MeJA, respectively. These values were 1.79, 1.60, and 1.13 times higher than the control. It indicated that barley seedlings attenuate adversity injury by regulating POD, SOD, and CAT activities.

MeJA stress significantly up-regulated the expression of *HvCAT* and *HvSOD* in four-day-old seedlings (Figure 5D,E, *p* < 0.05), which were 1.27 and 1.26 times higher than in the control. At six days of germination, MeJA significantly up-regulated the expression of *HvCAT*, *HvSOD,* and *HvAPX* in four-day-old seedlings, 2.4-, 1.4-, and 2.5-fold the control. However, ZnSO_4_ treatment significantly increased the expression of the *HvCAT* and *HvAPX* genes in seedlings only on day six of germination. These results indicated that different stresses caused differences in antioxidant gene expression.

Furthermore, during barley germination, MeJA and ZnSO_4_ treatments significantly improved DPPH and ABTS clearance rates compared to the control (Figure 5G,H, *p* < 0.05). From Figure 5G, the DPPH clearance rate reached its maximum under the ZnSO_4_ treatment, which was 2.48- and 1.99-fold higher than the control, respectively. Maximum ABTS clearance was achieved in four-day-old seedlings under MeJA treatment, it was increased by 38.2% more than the control.

### 2.6. Effects of Treatments on the Expression of Calcium Target Protein Genes

**Figure 6:**
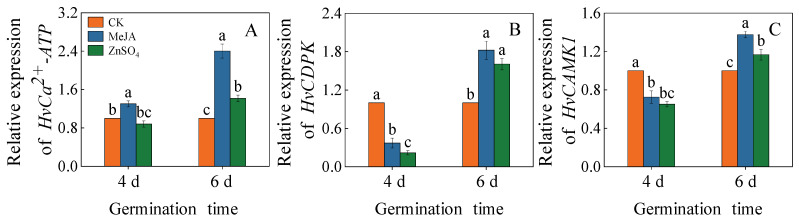
Effects of MeJA/ZnSO_4_ on the gene expression levels of *HvCa^2+^-ATP* (**A**), *HvCDPK* (**B**), and *HvCAMK1* (**C**) of barley seedlings. Lowercase letters indicate significant differences (*p* < 0.05) between treatments for the same germination time.

In the fourth barley seedling, the gene expression level of *calcium-adenosine triphosphate (HvCa^2+^-ATP)* under the MeJA treatment was significantly up-regulated (Figure 6A, *p* < 0.05), which was 1.30 times higher than the control. However, compared to the control, the gene expression levels of *calcium-dependent protein kinase* (*HvCDPK*) and *calmodulin-dependent protein kinase-related kinase* (*HvCAMK1)* had no discernible change under the MeJA treatment on the fourth day (Figure 6A–C). On the six-day-old barley seedlings, compared to the control, the MeJA and ZnSO_4_ treatments significantly up-regulated the gene expression levels of *HvCa^2+^-ATP*, *HvCAMK1*, and *HvCDPK*.

## 3. Discussion

The germination process of seeds involves a series of morphological and physiological changes, which is the most vigorous period of higher plant life activities, and the germination process is easily disturbed by environmental factors. The study showed that the application of 100 μM MeJA and 4 mM ZnSO_4_ significantly inhibits the growth of barley seedlings (Figure 1A,B), disrupts the water balance, and causes a decrease in the osmotic regulation capacity. MDA serves as the end product of membrane lipid peroxidation, and its content reflects the degree of membrane lipid peroxidation and the strength of the response to adverse conditions. This study found that MeJA and ZnSO_4_ treatment significantly increased the MDA content in seedlings (Figure 3). The result indicated that under stress conditions, plants have difficulty with water and nutrient absorption and are subject to osmotic stress, affecting the protein and phospholipid bilayer structure of the membrane system, thereby reducing the stability of the biological membrane and causing growth damage to barley seedlings. Under adverse conditions, plants can accumulate a large amount of reactive oxygen species (ROS), leading to oxidative damage to plants. In the future, we will determine the content of hydrogen peroxide and superoxide anions using the Pasternak methods [19], and we will use fluorescence microscopy to locate hydrogen peroxide and superoxide ions in barley seedlings, further clarifying the oxidative harm induced by MeJA and ZnSO_4_ stress in barley seedlings.

Controlling specific germination conditions can double the enrichment of specific functional components, including phenolic acid [20,21], to improve the nutritional value of plant seed food. Studies have shown that adversity stress can activate a series of physiological, biochemical, and molecular regulatory mechanisms in plants, leading to the activation of enzymes and genes involved in stress tolerance mechanisms to ensure normal plant growth and development. Phenolic acid is not only an important secondary metabolite in plants, but also an important member of the non-enzymatic protection system. Most phenolics are usually produced by the metabolic pathway of phenylpropanoid and are stimulated by biotic and abiotic stresses [22]. In this study, barley seedlings treated with MeJA and ZnSO_4_ activated the phenylpropanoid metabolic pathway and promoted the accumulation of total phenolic acid (Figure 2). The radical scavenging capacity of ABTS and DPPH responds to a greater extent to the antioxidant capacity of phenolics. In this study, it was found that MeJA and ZnSO_4_ treatments significantly increased ABTS and DPPH scavenging (Figure 5G,H), and ABTS and DPPH scavenging were highly correlated with the total phenolic acid content. This is in agreement with the previous findings of Kim et al. [23]. These findings indicated that the accumulation of phenolic acid is an important condition for improving the antioxidant capacity.

The increase in phenolic content was associated with changes in gene expression and the viability of key enzymes of the phenolic synthesis pathway. PAL and C4H enzymes play a crucial role in the synthesis of phenolic acid [24]. In this study, the application of MeJA significantly promoted *HvPAL* and *HvC4H* gene expression and enhanced PAL and C4H activities in six-day-old seedlings (Figure 5A–D). ZnSO_4_ treatment significantly promoted *HvC4H* expression and enhanced C4H activity in six-day-old seedlings (Figure 5B,D). This outcome suggested that both ZnSO_4_ and MeJA treatments can increase C4H activity by upregulating *HvC4H* transcription, thus promoting phenolic acid accumulation. This is consistent with the findings of Ma [25], who noted that the increase in phenolic acid content was positively correlated with PAL, C4H, and 4CL activity. In addition, Wang [26] showed that NaCl treatment promoted the gene expression of *HvPAL*, *HvC4H*, *HvC3H*, *Hv4CL*, and *HvCOMT* and enhanced the activities of PAL and C4H, which in turn promoted phenolic acid enrichment. In this study, we found that MeJA treatment significantly increased the expression of *Hv4CL*, *HvC3H*, and *HvF5H* in six-day-old seedlings. ZnSO_4_ treatment resulted in the significant up-regulation of *Hv4CL* and *HvCOMT* expressions in six-day-old seedlings. This result indicated that MeJA and ZnSO_4_ treatments promoted phenolic acid synthesis by up-regulating phenolic acid metabolism-related genes, but the same genes were expressed differently under different treatments.

In addition to phenolic acid, antioxidant enzymes also play a role in mitigating growth damage caused by adverse conditions. They can effectively respond to the membrane damage caused by adverse stresses in barley seedlings together with phenolic acid, thus ensuring the normal growth of plants. In this study, the changes in the activities of three antioxidant enzymes (APX, CAT, and SOD) induced by MeJA and ZnSO_4_ treatments were investigated, among which CAT and APX showed the greatest increase in activity, and the activities of enzymes were significantly increased to different degrees. Perveen et al. [27] reported an increase in the activities of CAT, POD, and APX, as well as an increase in the phenolic acid content, in barley seedlings treated with ZnSO_4_. In a study on *Panax ginseng*, the addition of MeJA increased the antioxidant capacity and phenolic content [28]. These results suggested that the accumulation of phenolic acid and the increase in the activities of antioxidant enzymes enhanced the antioxidant properties of barley seedlings.

Ca^2+^ serves as a second messenger in response to various abiotic stresses. Ca^2+^ floods into the cell membrane through Ca^2+^ channels [29]. Calmodulin and CDPK are two calcium-binding proteins that play a role in calcium signaling during the stress response [30]. According to the report, an increase in intracellular Ca^2+^ levels could affect the phenolic acid content, and the application of the Ca^2+^ inhibitor LaCl_3_ and protein kinase antagonists could reduce phenolic acid levels [31]. This study observed that treatments with MeJA and ZnSO_4_ up-regulated the relative expression of calmodulin-related genes, including *HvCa^2+^-ATP*, *HvCAMK*, and *HvCDPK* (Figure 6). These results suggested that MeJA and ZnSO_4_ treatments increased Ca^2+^ release. In the future, we will investigate the study of Ca^2+^-mediated phenolic acid synthesis under MeJA and ZnSO_4_ treatments.

## 4. Materials and Methods

### 4.1. Materials and Experimental Design

The barley seeds, provided in 2020 by the Jiangsu Provincial Academy of Agricultural Sciences, were stored at −20 °C. The fully seeded barley was weighed, rinsed in distilled water, and disinfected in a 0.5% (*v*/*v*) aqueous sodium hypochlorite solution for 15 min. After disinfection, the barley was immersed in three times its volume of deionized water and soaked in a light-protected water bath at 25 °C for 6 h. The soaked barley was placed in separate germination trays and then incubated at 25 °C protected from light. Each treatment was sprayed with 40 mL of different solutions every 12 h during germination. (1) CK: The barley was sprayed with distilled water; (2) MeJA treatment: The barley was sprayed with 100 μM MeJA; (3) ZnSO_4_ treatment: The barley was sprayed with 4 mM ZnSO_4_. The selected MeJA and ZnSO_4_ concentrations were optimized according to the pre-experiment (Supplement Figures S2 and S3). The seedlings were sampled during germination after four and six days for analysis.

### 4.2. Seedling Length, Fresh Weight, Dry Weight, and Growth Status

Thirty seedlings were randomly selected for each treatment, and their length, fresh weight, and dry weight were measured according to Ma et al. [25].

### 4.3. Measurement of Total Phenolic Acid and Total Phenolic Content

The total phenolic and total phenolic acid contents were determined according to the method of Wang et al. [22]. Specifically, barley seedlings were homogenized with 50% methanol to obtain a homogenate. The homogenate was centrifuged to obtain the supernatant. The supernatant was thoroughly mixed with Folin-phenolic and sodium carbonate and then incubated at 25 °C for two hours in the dark. The absorbance value was measured at 765 nm, and the total phenol content was calculated based on the standard gallic acid curve.

Furthermore, the supernatant was mixed with 50% methanol, 0.3% sodium dodecyl sulfate, and 0.6% ferric chloride, then thoroughly mixed and placed in the dark for 5 min. The absorbance at 760 nm was read to determine the total phenolic acid content.

### 4.4. Measurement of the Activity of Enzymes for Phenolic Acid Metabolism

The PAL and C4H activities were detected using the method described by Ma et al. [32]. Specifically, barley seedlings were homogenized at low temperatures with an extraction buffer. The resulting homogenate was centrifuged, and the supernatant was isolated. The activities of PAL and C4H were calculated from the absorbance of the supernatant at 290 nm and 340 nm, respectively, with a change of 0.01 per minute.

### 4.5. Measurement of MDA Content

The MDA content was measured using the methodology described by Zhuang et al. [33]. Barley seedlings were ground with TCA. After centrifugation (10,000× *g*, 20 min), the supernatant was boiled with TBA for 20 min. The absorbance values of the supernatant were determined at 450 nm, 532 nm, and 600 nm, respectively.

### 4.6. Measurement of Antioxidant Enzyme Activity, DPPH, and ABTS

The POD, CAT, and APX activities were determined according to Chen et al. [34]. The SOD activity was measured following the instructions of Sudhakar et al. [35]. Barley seedlings were ground in an ice bath containing sodium phosphoric acid buffer (pH 7.0, 50 mM). The suspensions were centrifuged (10,000× *g*, 4 °C, 15 min), and the supernatant was used to measure the activity of the antioxidant enzymes. A change of 0.01 at OD_290 nm_ per minute was expressed as one unit of APX activity. A change of 0.01 at OD_240 nm_ per minute was expressed as one unit of CAT activity. A change of 0.01 at OD_560 nm_ per minute was expressed as one unit of SOD activity.

The DPPH and ABTS assays were conducted following the method by Rumpf et al. [36]. The absorbance at 734 nm was read on a spectrophotometer to determine the ABTS scavenging activity. The DPPH scavenging activity was calculated by measuring the absorbance at 515 nm using vitamin C as a positive control.

### 4.7. Measurement of Soluble Protein and Free Amino Acid Content

The free amino acid content and the soluble protein content were measured according to the instructions of Yin et al. [37]. Barley seedlings were ground with acetic acid, and the supernatant was obtained via centrifugation. The supernatant was mixed with ninhydrin and ascorbic acid and then heated in boiling water. The absorbance of the sample at 570 nm was determined. The soluble proteins were determined using Thomas Brilliant Blue. Barley seedlings were taken and ground well by adding PBS. The homogenate was centrifuged, and the supernatant was separated. The supernatant and the calmer brilliant blue G-250 solution were mixed thoroughly, and then, the absorbance was colorimetrically measured at 595 nm.

### 4.8. Quantitative Real-Time PCR Analysis and RNA Extraction

Fresh seedlings were germinated for four and six days before being washed and frozen in liquid nitrogen. Liquid nitrogen was used to grind the seedlings. Total RNA from barley seedlings was obtained using an RNA extraction kit (RC401-01, Vazyme, Nanjing, China). A reverse transcription kit (R323-01, Vazyme, Nanjing, China) is used to synthesize cDNA from total RNA. Samples were analyzed for fluorescence using the SYBR (Vazyme, Nanjing, China). Table S1 shows the primers used in this study. Relative gene expression levels were calculated using the 2^−ΔΔCt^ method.

### 4.9. Statistical Analysis

All experiments were performed with at least three biological replicates (*n* ≥ 3). Results were presented as means ± standard deviation. Analysis of variance was conducted using SPSS 22.0 software (SPSS Inc., Chicago, IL, USA). Significant differences were analyzed using the Tukey test at a level of *p* < 0.05.

## 5. Conclusions

The above results showed that both 100 μM MeJA and 4 mM ZnSO_4_ treatments inhibited the growth of barley seedlings, as evidenced by the shortening of seedling length and the accumulation of MDA. As a countermeasure, MeJA and ZnSO_4_ treatments enhanced the antioxidant capacity of barley sprouts through related enzyme activities (CAT, SOD, and APX) and gene expression. In addition, MeJA and ZnSO_4_ treatments increased the synthesis of the phenolic acid content in barley seedlings by increasing PAL and C4H activities. This study revealed that MeJA and ZnSO_4_ treatments effectively enhanced phenolic acid synthesis in barley seedlings and provided a theoretical basis for secondary metabolites in plants.

## Data Availability

The data presented in this study are available on request from the corresponding author.

## Supplementary Materials

The following supporting information can be downloaded at: https://www.mdpi.com/article/10.3390/plants13111512/s1, Table S1: Sequence-specific primers used in the present study; Figure S1: Effect of different treatments on phenolic acid content of barley seedlings; Figure S2: Effect of MeJA concentration on growth performance (A), phenolic acid content (B), and length (C) of barley seedlings; Figure S3: Effect of ZnSO_4_ concentration on growth performance (A), phenolic acid content (B), and length (C) of barley seedlings.
