# Supplementary material for: Methyl Jasmonate and Zinc Sulfate Induce Secondary Metabolism and Phenolic Acid Biosynthesis in Barley Seedlings
Source: Plants (Basel). 2024 May 30;13(11):1512. doi: 10.3390/plants13111512 (PMC11174577; doi:10.3390/plants13111512)
Supplement: Supplementary file 1 [file plants-13-01512-s001.zip › plants-2976980-supplementary.pdf]

**SUPPLEMENT FIGURE CAPTIONS**

**Figure S1.** Effect of different treatments on phenolic acid content of barley seedlings. Lowercase letters indicate significant ($p < 0.05$) differences between treatments for the same germination time. CK: Control; UV-B: 9 h/24 h Ultraviolet-B; MeJA: 100 μM methyl jasmonate; $ZnSO_4$: 4 mM zinc sulfate; GA: 200 μM gibberellic acid; Heat: 45℃ 30 min/d.

**Figure S2.** Effect of MeJA concentration on growth performance (A), phenolic acid content (B), and length (C) of barley seedlings. Lowercase letters indicate significant ($p < 0.05$) differences between treatments for the same germination time. CK: Control; $M_1$: 50 μM MeJA; $M_2$: 100 μM MeJA; $M_3$: 150 μM MeJA; $M_4$: 200 μM MeJA.

**Figure S3.** Effect of $ZnSO_4$ concentration on growth performance (A), phenolic acid content (B), and length (C) of barley seedlings. Lowercase letters indicate significant ($p < 0.05$) differences between treatments for the same germination time. CK: Control; $Z_1$: 2 mM $ZnSO_4$; $Z_2$: 4 mM $ZnSO_4$; $Z_3$: 6 mM $ZnSO_4$; $Z_4$: 8 mM $ZnSO_4$.

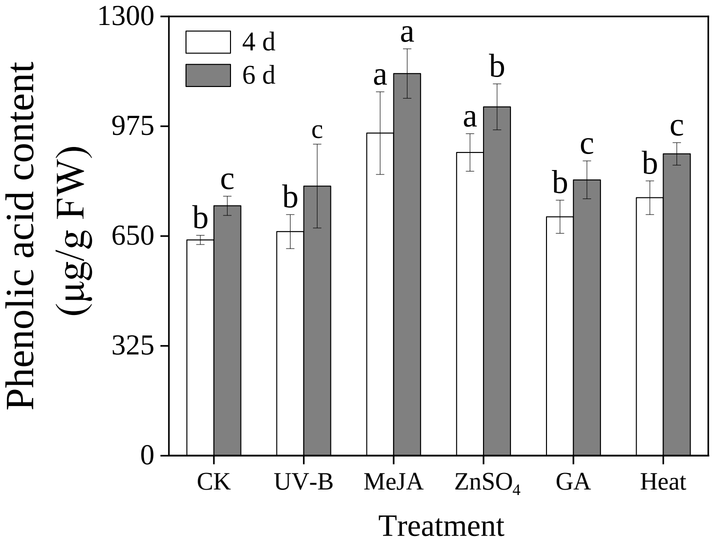
Phenolic acid content
(μg/g FW)
1300
975
650
325
0
4 d
6 d
a
a
b
a
c
c
b
b
c
b
b
c
CK
UV-B
MeJA
ZnSO4
GA
Heat
Treatment

**Figure S1**

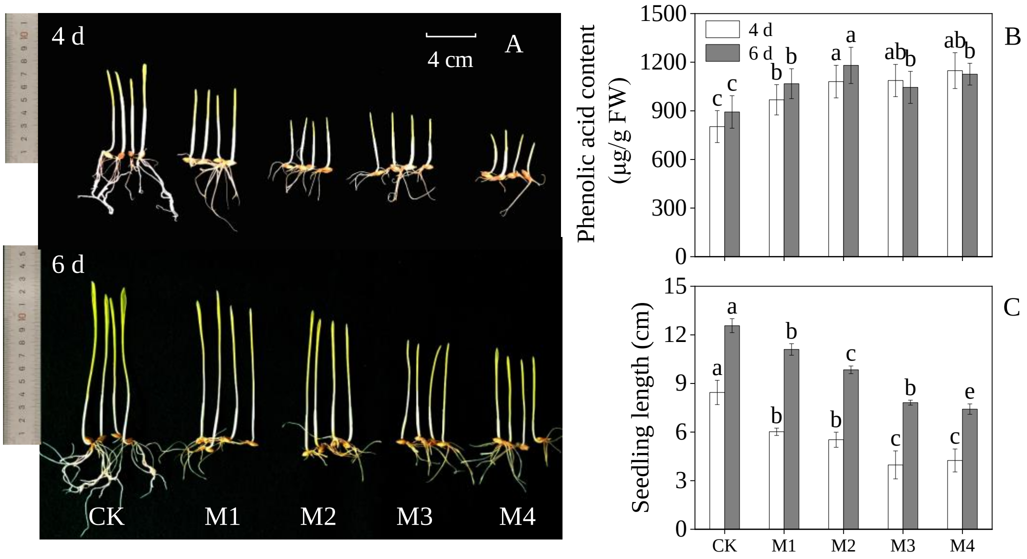
4 d
4 cm
A
6 d
CK
M1
M2
M3
M4
Phenolic acid content (μg/g FW)
B
Seedling length (cm)
C

**Figure S2**

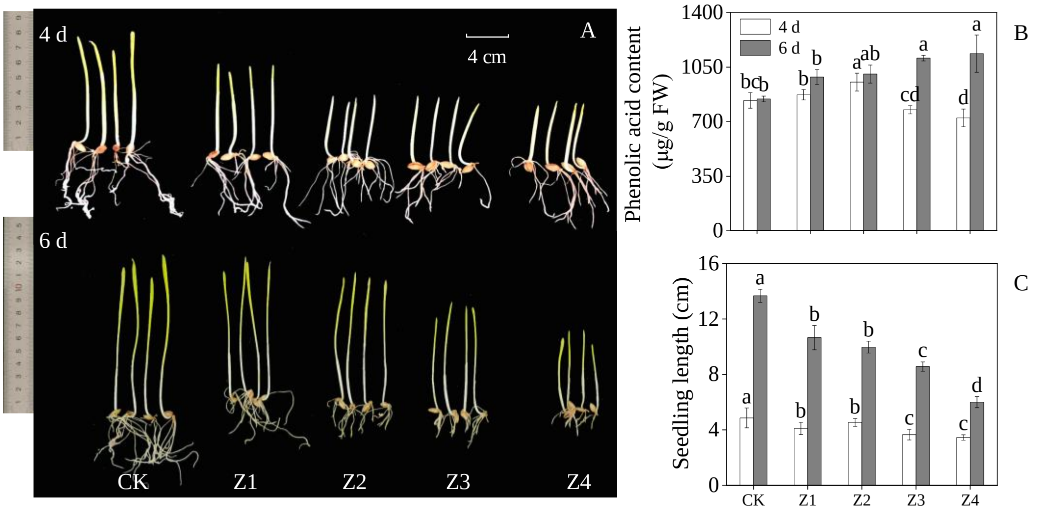
4 d
6 d
4 cm
A
CK
Z1
Z2
Z3
Z4
B
Phenolic acid content (μg/g FW)
4 d
6 d
1400
1050
700
350
0
C
Seedling length (cm)
16
12
8
4
0

**Figure S3**

**Supplement Table S1.** Sequence-specific primers used in the present study.

| Gene name | Forward Primer (5' to 3') | Reverse Primer (5' to 3') |
|---|---|---|
| *HvPAL* | CACTGAATGCCGATCATACCC | CCGTTCCAACCCTTGAGACA |
| *HvC4H* | AGCTCGCCGCCTACAACATC | GCCTCCACGTGCTTCTCCTC |
| *Hv4CL* | GGTGGAGATCGCCAAGAGCC | CTCCGTCATCCCGTACCCCT |
| *HvC3H* | ATCACCGCTGGGATGGACA | GGTACGGCAGGTTCTGGAAGT |
| *HvCOMT* | GCCGTCAAAGGCATCAACT | GCAAGGCGTCATAGCAGTTC |
| *HvF5H* | TCGACGACATGCTCGCCTTC | TCCCGCCAAACATCACGTCC |
| *HvCAT* | TGGACGGATGGTACTGAACA | GTGCCTTTGGGTATCAGCAT |
| *HvAPX* | CGCCCTCTTGTGGAGAAATA | CGCGCATAGTAGCAGCAGTA |
| *HvSOD* | CCCCTCACCAAGTCAGTCAT | ATTGCAAGTCGGTGTCCTTC |
| *HvCa$^{2+}$-ATP* | CCACCGTCATCTGCTCCGAC | ATTGCACACAGCTGCGACCT |
| *HvCAMK1* | CGGTGTCCTTCATCTTGCG | ACGAGGTTGATGCTGACGG |
| *HvCDPK* | TTTGCTATGTGGTGTCCCCC | GCAGAGAGCCGCTTTGTAGG |
| *HvActin* | TCGTGAGAAGATGACCCAGA | CCGAGTCCAGCACAATACCT |
